# Persistence and safety of subcutaneous infliximab up to 1 year after switching from intravenous infliximab in pediatric inflammatory bowel disease: a multicenter real-world cohort study

**DOI:** 10.1093/ibd/izaf335

**Published:** 2026-02-01

**Authors:** Esmée Helen Boute, Laura Gianolio, Shaden Mahmmod, Saverio Pochesci, Katherine Armstrong, Paul Henderson, David Charles Wilson, Lissy de Ridder, Richard Kay Russell, Johanna Caroline Escher

**Affiliations:** Department of Paediatric Gastroenterology, Erasmus Medical Centre Sophia Children’s Hospital, Rotterdam, The Netherlands; Department of Paediatric Gastroenterology, Royal Hospital for Children and Young People, Edinburgh, Scotland; Department of Paediatric Gastroenterology, Erasmus Medical Centre Sophia Children’s Hospital, Rotterdam, The Netherlands; Department of Paediatrics, University Medical Centre Utrecht, Utrecht University, Utrecht, The Netherlands; Department of Paediatric Gastroenterology, Royal Hospital for Children and Young People, Edinburgh, Scotland; Department of Paediatric Gastroenterology, Royal Hospital for Children and Young People, Edinburgh, Scotland; Department of Paediatric Gastroenterology, Royal Hospital for Children and Young People, Edinburgh, Scotland; Child Life and Health, Centre of Inflammation Research, University of Edinburgh, Edinburgh, Scotland; Department of Paediatric Gastroenterology, Royal Hospital for Children and Young People, Edinburgh, Scotland; Child Life and Health, Centre of Inflammation Research, University of Edinburgh, Edinburgh, Scotland; Department of Paediatric Gastroenterology, Erasmus Medical Centre Sophia Children’s Hospital, Rotterdam, The Netherlands; Department of Paediatrics, Willem-Alexander Children’s Hospital/Leiden University Medical Centre, Leiden, The Netherlands; Department of Paediatric Gastroenterology, Royal Hospital for Children and Young People, Edinburgh, Scotland; Child Life and Health, Centre of Inflammation Research, University of Edinburgh, Edinburgh, Scotland; Department of Paediatric Gastroenterology, Erasmus Medical Centre Sophia Children’s Hospital, Rotterdam, The Netherlands

**Keywords:** pediatric, Crohn disease, ulcerative colitis, subcutaneous infliximab, real-world cohort

## Abstract

**Introduction:**

Real-world data regarding subcutaneous infliximab (SC-IFX) in patients with pediatric inflammatory bowel disease IBD (PIBD) is scarce. We evaluated SC-IFX as maintenance therapy in PIBD patients who switched to SC-IFX from intravenous infliximab (IV-IFX) treatment.

**Methods:**

In this retrospective multicenter study we identified PIBD patients who switched to SC-IFX. The primary outcome was treatment persistence at up to 12 months post-switch. Secondary outcomes included relapse rate (defined as Pediatric Ulcerative Colitis Activity Index [PUCAI]  ≥10/ weighted PCDAI  ≥ 12.5 with biochemical/endoscopic evidence of disease activity), IFX trough levels, immunogenicity, safety, and acceptance.

**Results:**

Sixty-six patients switched to SC-IFX (48% males; median switch-age, 16.5 years; IQR, 14.9-17.3 years; median switch-weight, 60 kg; range, 13-102 kg), 41/66 (62%) with Crohn Disease. Pre-switch, the median IV-IFX maintenance dose was 10 mg/kg every 6 weeks; 58/66 patients (88%) were in clinical remission. The initial SC-IFX regimen was 120 mg every other week in 62/66 patients (94%). SC-IFX persistence was 78% (95% CI, 66-91) at 12 months post-switch, with 89% of patients persisting on IFX, either intravenous (IV) or subcutaneous (SC), at the end of follow-up. Relapses were observed in 11/66 patients (17%) over a median follow-up of 11.0 months (IQR, 5.1-12.0); 6 patients underwent SC-IFX dose intensification, with 3 successfully regaining clinical response. Regarding anti-drug antibodies (ADA), 3 out of 4 patients who were ADA positive on IV-IFX resolved post-switch. Overall, 19/66 patients (29%) reported 21 adverse events (AEs), including 3/21 severe AEs. The majority (53/66 patients; 80%) expressed a positive attitude toward SC-IFX.

**Conclusions:**

The largest documented PIBD cohort switching to SC-IFX to date showed high treatment persistence at 1 year, confirming SC-IFX as an effective and safe maintenance alternative to IV-IFX.

## Graphical abstract

**Graphical Abstract:**
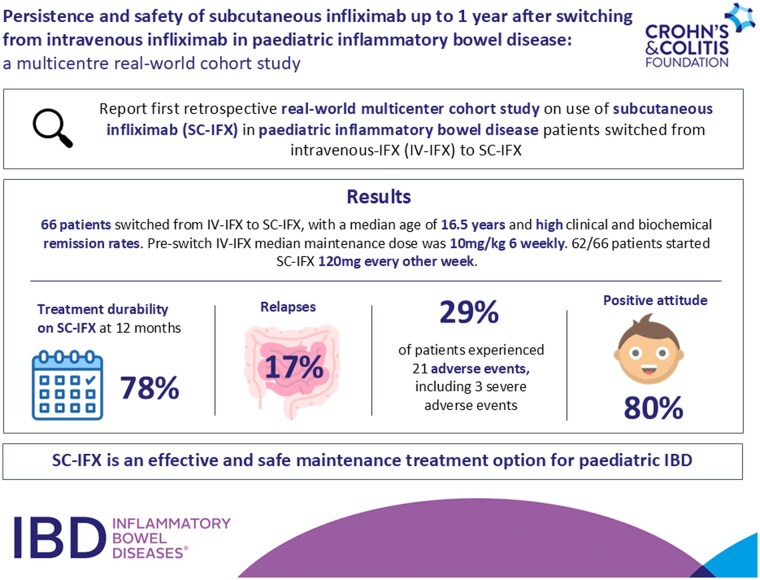

Key Messages *What is already known?*

Subcutaneous infliximab (SC-IFX) has established efficacy and safety in the treatment of adult inflammatory bowel disease (IBD), however its role in pediatric IBD (PIBD) remains largely unknown.

*What is new here?*

This real-world study, analyzing the largest to date documented PIBD cohort of patients switched from IV-IFX to SC-IFX, showed high treatment persistence and acceptance, and the favorable effectiveness and safety profiles of SC-IFX.

*How can this study help patient care?*

We suggest that SC-IFX is an effective and safe maintenance treatment alternative to IV-IFX in PIBD, with potential for broader clinical use and additional pharmacokinetic studies warranted in younger patients.

## Introduction

Infliximab (IFX), a chimeric IgG1 monoclonal antibody targeting tumor necrosis factor alpha (TNFα), has dramatically changed the treatment landscape in inflammatory bowel disease (IBD), since its initial approval for adult use by the European Medicines Agency (EMA) in 1998.^1^ In pediatric medicine IFX has been established as the first-line biologic in the treatment of ulcerative colitis (UC) and as a first-line biologic option, along with adalimumab, in Crohn disease (CD).^2^^,^^3^

Traditionally, IFX has been administered intravenously as a weight-based regimen for both induction and maintenance therapy. While intravenous IFX (IV-IFX) is highly effective in both CD and UC, the need for regular hospital visits, venous cannulation, and time-consuming infusions reduces its acceptability. In contrast, subcutaneous administration offers practical advantages, being generally preferred by patients for its convenience and lower impact on daily life.^4^

In 2020, the European Medicines Agency (EMA) approved the subcutaneous (SC) formulation of CT-P13 infliximab biosimilar (SC-IFX, CT-P13; Remsima—Celltrion Healthcare, Incheon, South Korea) for maintenance therapy in adult IBD patients, with a standard dosing regimen of 120 mg every other week following at least 2 intravenous induction infusions.^5^

Emerging adult data from randomized controlled trials (RCTs) and real-world studies have shown that SC-IFX provides comparable efficacy and safety to IV-IFX, with a potentially superior pharmacokinetic profile characterized by lower immunogenicity and stable, consistently higher serum drug levels.^6–17^ Two recent meta-analyses further support these findings, suggesting comparable efficacy of SC and IV administration with indications of potential additional clinical advantages, warranting further investigation.^12^^,^^18^

Considering the specifics of the pediatric population, dedicated evidence is needed; however, at present no specific pediatric clinical trials have been published.^19^ In pediatric practice, 2 single center case series have been published to date, in 2023 and 2025, involving 7 and 21 patients, respectively.^20^^,^^21^ The aim of the present study was to describe a large cohort of pediatric IBD (PIBD) patients switched from IV-IFX to SC-IFX, and assess SC-IFX persistence, effectiveness, safety, and acceptability after switching.

## Methods

### Study design and patient cohort

This retrospective multicenter cohort study was conducted at 2 IBD referral centers (Erasmus Medical Centre-Sophia Children’s Hospital in Rotterdam, The Netherlands and Royal Hospital for Children and Young People in Edinburgh, Scotland). Seven patients included in the Edinburgh cohort were also described in a case-series by Gianolio et al.^20^ Pediatric patients with IBD, previously treated with IV-IFX and switched to SC-IFX as maintenance therapy at the discretion of the treating clinician between January 2021 and February 2025 were included with follow-up until June 2025. PIBD was diagnosed as per the revised Porto criteria.^22^ Disease phenotype was defined according to the Paris Classification.^23^ Patients with IBD unclassified (IBD-U) were grouped with UC patients for analyses.

### Data collection

Patient data were extracted from the electronic health records using standardized case report forms in the participating centers at the time of the switch to SC-IFX (baseline) and at 2, 6, and 12 months after switching, when available. All patients in the Edinburgh cohort were initially switched to SC-IFX (Remsima) 120 mg every other week (EOW), while in the Rotterdam cohort SC-IFX was prescribed either 120 mg EOW or 120 mg every week (EW) at clinician discretion, taking IV-IFX dosing into account.

At baseline, the following data were collected: age; sex; height; body weight; disease type; disease phenotype; previous medical treatments and surgery; concomitant medical treatments; IV-IFX treatment duration, dose, and interval; clinical disease activity (the Paediatric Ulcerative Colitis Activity Index [PUCAI] score for UC and IBD-U^24^ and the weighted Paediatric Crohn’s Disease Activity Index [wPCDAI] score for CD^24^); fecal calprotectin (FC); serum C-reactive protein (CRP); erythrocyte sedimentation rate (ESR); and albumin and IFX trough levels with anti-drug antibodies (ADAs) pre-switch. Baseline IV-IFX trough levels were recorded up to 1 year before switch, if dose and interval remained unchanged. The intensified IV-IFX regimen was defined as infusions administered at a dose of 10 mg/kg 4 times weekly. Additionally, the reasons for switching from IV-IFX to SC-IFX were documented, and the distance from patient’s home address to the hospital was recorded. At each follow-up timepoint clinical and laboratory data were collected, including disease activity, concomitant medications changes, SC-IFX regimen adjustments, adverse events (AEs), FC, CRP, ESR, albumin, and IFX levels with ADA detection. Lastly, patient feedback in terms of treatment acceptance and satisfaction was sought informally at follow-up visits. When patients reported any form of dissatisfaction during a visit, including pain or discomfort, or if it could not be inferred retrospectively, this event was categorized as patient dissatisfaction.

Standard cutoffs were used to define categories of disease activity. Specifically, in CD, clinical remission was defined by a wPCDAI score of < 2.5 and in UC and IBD-U by a PUCAI score of < 10. Biochemical disease activity was defined as FC > 250 mcg/g; and biochemical remission as FC <250 mcg/g. In patients with CD, relapse was defined as wPCDAI ≥ 12.5 and FC > 250 mcg/g or as recurrence of perianal or endoscopic disease activity defined as a SES-CD score of > 2 with ulcers. In patients with UC, relapse was defined as PUCAI ≥ 10 and FC > 250 mcg/g or endoscopic disease activity defined as an endoscopic Mayo score of > 0. Loss of response (LOR) was defined as the loss of therapeutic effectiveness during maintenance treatment in patients who had initially responded to IFX induction therapy, leading to therapeutic adjustments. Immunogenic LOR was defined as low (<5 µg/mL) or undetectable IFX (trough) levels accompanied by high ADA (>100 ng/mL) in a patient previously in remission on IFX, ultimately requiring discontinuation of IFX.

### IFX levels and antibodies

Serum IFX (trough) levels and ADA were quantitatively measured using enzyme-linked immunosorbent assay (ELISA) (Promonitor assay [Grifols] in Edinburgh; LISA TRACKER assay (Theradiag) in Rotterdam). The upper detection limits for IFX levels were 14.0 µg/mL until January 2024 and thereafter 12.0 µg/mL for the Promonitor assay and 20.0 µg/mL for the LISA TRACKER assay. Values at the upper limit of detection were approximated as 12.0, 14.0, or 20.0 µg/mL, respectively, for the purpose of analysis. Measurements above these thresholds were only performed following specific requests from the treating physician. ADA testing was automatically performed by the laboratory when IFX levels were <7.0 µg/mL in Edinburgh and <1.0 µg/mL in Rotterdam or upon specific request by the treating physician.

### Economic and environmental impact

The economic impact was estimated by calculating both direct costs (including IV-IFX and SC-IFX costs, non–drug administration costs, and Diagnosis Treatment Combination [DBC]) and indirect costs (such as occupational and travel expenses), based on the median dosing regimen of our cohort. The environmental impact was assessed in terms of kilometers saved and the corresponding reduction in greenhouse gas emissions. A detailed description of the methodology used for the economic and environmental analyses—adapted from a previously published study—is provided in Supplemental Content 1.^20^

### Outcomes

The primary outcome was the SC-IFX persistence rate at up to 12 months post-switch from IV-IFX. Persistence was defined as the proportion of patients remaining on SC-IFX after the switch. Secondary outcomes included evaluation of (1) changes in clinical (PUCAI/wPCDAI) and biochemical (FC, CRP, and ESR) disease activity, (2) changes in serum IFX levels and immunogenicity before and after switch to SC-IFX, (3) trends in co-immunosuppression association after SC-IFX switch and its impact on disease activity, (4) rates of AEs and severe AEs post-switch to SC-IFX, (5) predictors of SC-IFX treatment persistence and relapse, (6) economic and environmental impact of SC-IFX compared to IV-IFX and (7) SC-IFX patient acceptance and satisfaction regarding the switch.

### Statistical analysis

Descriptive statistics were used to analyze patient characteristics. Continuous variables were presented as mean ± SD for normally distributed data, or median and IQR for non-normally distributed data. The assumption of normality was examined using histograms and quantile-quantile plots and assessed using the Shapiro-Wilk test. The unpaired t-test and the Mann Whitney U test were used to compare continuous variables between groups, depending on the distribution of the data. Categorical variables were presented as frequency (%) and compared using the Chi squared test or Fisher exact test. The Wilcoxon signed rank test was used to compare differences across medians for paired non-normally distributed data. Kaplan-Meier survival analysis was used to assess treatment persistence. Finally, we performed an exploratory Cox Proportional Hazards regression to assess variables associated with the occurrence of relapse and treatment persistence. Variables were chosen on the basis of clinical relevance. Missing data for predictor variables were imputed using the mice package in R. *P* values of <.05 were considered statistically significant. All analyses were carried out using R software (Version 2023.12.1).

### Ethical considerations

This study was approved by the local ethics committee of the Erasmus Medical Centre for the Rotterdam cohort (ethics approval number MEC-2018-1187). In line with local protocol, formal ethical approval was not required in Edinburgh as this was a review of service delivery and clinical practice. Written informed consent was obtained from participants aged 12 years and above, as well as from the guardians of participants aged younger than 16 years in the Rotterdam cohort. As an audit of clinical practice, informed consent was not required in the Edinburgh cohort, in accordance with local ethics committee guidance.

## Results

### Baseline characteristics

A total of 66 patients (48% male) were switched to SC-IFX at a median age of 16.5 years (IQR, 14.9-17.3 years). Of these, 41 patients (62%) had CD, 22 (33%) UC, and 3 (5%) IBD-U. Detailed data on disease phenotype, behavior, and prior therapies are presented in Table 1; baseline differences between the Edinburgh and Rotterdam cohort are provided in Table S1 in the Supplementary Material. In 35 patients (53%), switching from IV-IFX to SC-IFX was initiated by the treating pediatric gastroenterologist, after shared decision making with patients and parents. Among the remaining cases, the switch was prompted by patient- or parent-related factors: lifestyle issues in 17 patients (26%), problems with IV access in 11 patients (17%), and need to shorten IV-IFX interval in 3 patients (5%).

**Table 1:** Baseline characteristics of patients at time of initiation with subcutaneous infliximab.^a^

Characteristic	*n *= 66^a^
Diagnosis, *n* (%)	
Crohn Disease	41 (62%)
IBD Unclassified	3 (5%)
Ulcerative Colitis	22 (33%)
Sex, *n* (%)	
Female	34 (52%)
Male	32 (48%)
Weight at switch, kg, median (min, max)	60 (13, 102)
Weight, *z*-score, mean (SD)	0.43 (1.3)
Height, *z*-score, mean (SD)	−0.14 (1.0)
BMI at switch, mg/kg^2^, median (IQR)	20.7 (18.6, 24.0)
Disease duration at switch, mo, median (IQR)	34 (19, 51)
Age at switch, years, median (IQR)	16.50 (14.9, 17.3)
Paris age classification at diagnosis, CD, *n* (%)	
A1a	7 (17%)
A1b	34 (83%)
Paris disease behavior at diagnosis, CD, *n* (%)	
B1	34 (83%)
B2	4 (10%)
B3	3 (7%)
Paris disease location at diagnosis, CD, *n* (%)	
L1	5 (12%)
L2	7 (17%)
L3	28 (68%)
L4	1 (2%)
Paris upper disease location at diagnosis, CD, *n* (%)	
L4a	20 (49%)
L4b	2 (5%)
Growth restriction at diagnosis, CD, *n* (%)	
G0	35 (85%)
G1	6 (15%)
Perianal disease at diagnosis, yes, CD, *n* (%)	11 (27%)
Paris disease extent at diagnosis, UC, *n* (%)	
E1	1 (4%)
E2	4 (16%)
E3	6 (24%)
E4	14 (56%)
Disease severity at diagnosis, UC, *n* (%)	
S0	12 (48%)
S1	13 (52%)
Previous surgery, yes, *n* (%)	5 (8%)
Previous mesalazine, yes, *n* (%)	21 (32%)
Previous immunomodulator, yes, *n* (%)	61 (92%)
Previous corticosteroids, yes, *n* (%)	42 (64%)
Previous adalimumab, yes, *n* (%)	5 (8%)
Previous intravenous infliximab, yes, *n* (%)	66 (100%)
Duration intravenous infliximab therapy, mo, median (IQR)	22 (10, 36)
Intravenous infliximab interval, wk, *n* (%)	
4	18 (27%)
5	2 (3%)
6	30 (45%)
8	14 (21%)
Only induction scheme	2 (3%)
Intravenous infliximab dose, mg/kg, median (IQR)	10.0 (10.0, 10.0)
Intravenous infliximab regimen 10 mg/kg 4/wk, yes, *n* (%)	17 (26%)
Concomitant immunosuppression at switch, yes, *n* (%)	26 (39%)
Subcutaneous infliximab regimen, *n* (%)	
120 mg, 2/wk	62 (94%)
120 mg, 1/wk	4 (6%)
Clinical remission at switch, yes, *n* (%)	58 (88%)
wPCDAI at switch, median (IQR)	0 (0, 8)
Missing	0
PUCAI at switch, median (IQR)	0.0 (0.0, 0.0)
Missing	1
Fecal calprotectin at switch, µg/g, median (IQR)	46 (25, 123)
Missing	6
CRP at switch, mg/l, median (IQR)	1.0 (1.0, 1.1)
Missing	0
ESR at switch, mm/h, median (IQR)	8 (4, 14)
Missing	5
Albumin at switch, mm/h, median (IQR)	39.5 (37.0, 41.0)
Infliximab trough level on intravenous infliximab, µg/mL, median (IQR)	12.0 (9.4, 12.0)
Missing	7

Abbreviations: BMI, body mass index; CRP, C-reactive protein; ESR, erythrocyte sedimentation rate; IBD, inflammatory bowel disease; max, maximum; min, minimum; PUCAI, pediatric ulcerative colitis activity index; UC, ulcerative colitis; wPCDAI, weighted pediatric Crohn’s disease activity index.

a Data are *n* (%) or median (IQR) unless otherwise indicated.

After a median of 22 months (IQR 10-36 months) on IV-IFX, 62/66 patients (94%) were switched to the standard SC-IFX 120 mg EOW regimen, while 4 patients (6%) were initiated on 120 mg EW.

At the time of the switch, 26/66 patients (39%) were on stable concomitant immunosuppressive therapy, (azathioprine, 6-mercaptopurine, or methotrexate). The median IV-IFX regimen at the switch was 10 mg/kg every 6 weeks with 17/66 patients (26%) on a 10 mg/kg 4 weekly IV-IFX regimen. The median follow-up after switching was 11.0 months (IQR 5.1-12.0 months).

### Treatment persistence

**Figure 1:**
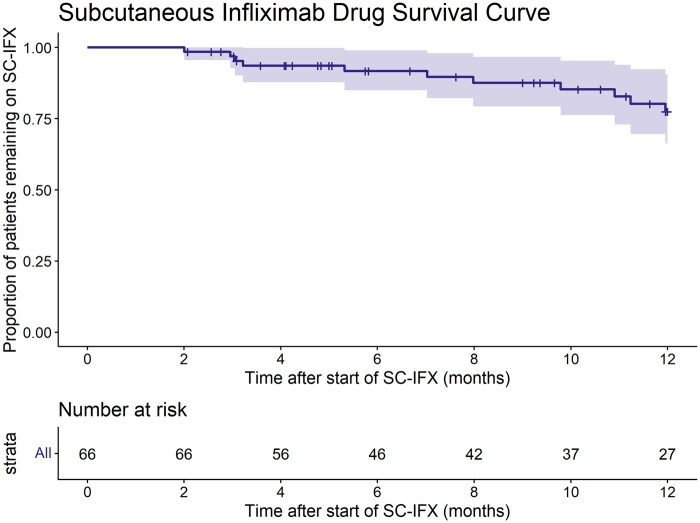
Kaplan-Meier curve of treatment persistence on subcutaneous infliximab.

At 12 months, the estimated SC-IFX discontinuation-free survival rate was 78% (95% CI, 66%-91%) (Figure 1), with no significant differences among IBD subtypes (Figure 1 and Supplementary Material). During follow-up, 11 patients (17%) discontinued SC-IFX treatment after a median of 7.0 months (IQR, 3.1-10.3 months). Reasons for discontinuation are detailed in Table 2 and were most commonly related to local adverse events (pain or swelling at the injection site, 6/11 patients, 55%), while LOR accounted for a smaller proportion (3/11 patients, 27%). Of the 11 patients who discontinued SC-IFX during the 1-year follow-up, 3 switched back to IV-IFX, 1 patient started mesalazine monotherapy as treatment de-escalation, 1 patient restarted SC-IFX (autonomous discontinuation), and 6 patients initiated a different biologic or small-molecule therapy, namely adalimumab, ustekinumab, tofacitinib, risankizumab, filgotinib, or upadacitinib. Of the 3 patients who switched back to IV-IFX, 2 patients remained in clinical and biochemical remission and 1 patient experienced a relapse, with adequate IFX trough levels. In total, 59/66 patients (89%) remained on IFX, either IV-IFX or SC-IFX, as their primary treatment target at last follow-up.

**Table 2:** Reasons for subcutaneous infliximab discontinuation.

Reason for SC-IFX discontinuation	Total no. = 11
Loss of response	3
Adverse events	6
Urticaria	1
Pain	5
Fear of injection	1
Own initiative	1

Abbreviation: SC-IFX, subcutaneous infliximab.

Several baseline clinical and biochemical variables were analyzed in univariate analysis with a bivariate model then constructed, incorporating body weight and prior treatment with a 10 mg/kg 4 weekly IV-IFX regimen, to assess their effect on treatment persistence. None of the variables analyzed were associated with treatment persistence (Table 3).

**Table 3:** Effect of variables on treatment persistence on SC-IFX and the occurrence of a relapse during SC-IFX treatment.

Predictor	Univariable		Multivariable	
	HR (95% CI)	*P* value	HR (95% CI)	*P* value
Predictors of treatment discontinuation on SC-IFX (total *n* = 66, *n* = 11 [16%] events)				
Weight at baseline, < 40 or > 40kg	0.29 (0.06-1.39)	.12	0.30 (0.06-1.46)	.14
Clinical disease score at baseline (log transformed)	1.23 (0.80-1.91)	.35		
FC at baseline (log transformed)	1.22 (0.74-2.00)	.39		
Coimmunosuppression at baseline, no/yes	2.04 (0.62-6.70)	0.24		
Diagnosis, CD/UC + IBD-U	1.03 (0.30-3.51)	0.97		
IV-IFX regimen 10 mg/kg, 4/wk, no/yes	2.13 (0.62-7.38)	0.23	2.08 (0.59-7.26)	0.25
­ Co-immunosuppression stop during follow-up, no/yes	1.04 (0.19-5.68)	0.97		
Predictors of the occurrence of a relapse during SC-IFX treatment (total *n* = 66, *n* = 11 [16%]) events				
Weight at baseline, kg	0.97 (0.93-1.01)	0.11		
Clinical disease score at baseline	1.05 (1.00-1.10)	0.04	1.05 (1.00-1.11)	0.04
Co-immunosuppression at baseline, no/yes	0.38 (0.08-1.78)	0.22	0.36 (0.08-1.72)	0.20
Diagnosis, CD/UC + IBD-U	0.64 (0.17-2.41)	0.51		
IV-IFX regimen 10 mg/kg, 4/wk no/yes	1.29 (0.34-4.91)	0.71		
Co-immunosuppression stop during follow-up, no/yes	2.00 (0.13-32.07)	0.62		

Abbreviation: CD, Crohn disease; HR, hazard ratio; IBD-U, inflammatory bowel disease unclassified; IV-IFX, intravenous infliximab; SC-IFX, subcutaneous infliximab. UC, ulcerative colitis.

### Disease relapses

A total of 11/66 patients (17%) experienced a disease relapse, of whom 8/11 had CD (73%) occurring after a median of 3.7 months from the SC-IFX switch (IQR, 1.5-7.2 months) (Table 4). Among these, 6 patients underwent treatment intensification through either dose escalation (240 mg EOW) or interval shortening (120 mg EW), resulting in regained clinical response in 3 patients (50%). Three patients switched to an alternative biologic/small molecule (ustekinumab, tofacitinib, and risankizumab) with 2 out of 3 previously failing an SC-IFX intensification. Additionally, 3 patients required initiation or escalation of concomitant therapies, including mesalazine dose increase (initiated alongside SC-IFX intensification) and 2 courses of antibiotics. No patients underwent intestinal surgery, although 1 patient developed a perianal fistula requiring seton placement. Follow-up data were unavailable for 2 relapsed patients due to transfer to adult care and loss to follow-up. Eight out of 11 patients who experienced a relapse had an IFX level while on SC-IFX, available before or during the relapse, all on a regimen of 120 mg EOW. The median IFX level was 14 µg/mL (IQR, 12-20) of whom 4/8 patients (50%) reached the ceiling of the corresponding assay. One patient had an IFX level below 10 µg/mL, namely 6.1 µg/mL.

**Table 4:** Adverse events during subcutaneous infliximab follow-up.^a^

Adverse events	No. (%)
Mild adverse event	
Pain/swelling at injection site	7 (10%)
Fatigue	2 (3%)
Infection	2 (3%)
Urticaria	1 (1%)
Appearance/worsening of eczema	6 (9%)
Serious adverse event	
IgA nephropathy requiring hospitalization	1 (1%)
Infection requiring hospitalization	1 (1%)
Fistula requiring seton drain	1 (1%)
Relapse	11 (17%)

a Data shown as number of events (percentage of total participants).

In univariate analysis, higher clinical scores at switch were positively associated with relapse risk over time (hazard ratio [HR], 1.05; 95% CI, 1.00-1.10; *P* = .04). The association with clinical scores remained significant in bivariate analysis (HR 1.05, 95% CI, 1.00-1.11; ***P* = 0.04**). No other factors including IBD subtype, weight at switch, concomitant immunosuppression at switch, co-immunosuppression stop after switch or 10 mg/kg 4 weekly IV-IFX regimen were significantly associated with relapse (Table 3).

### Clinical and biochemical trends

**Figure 2:**
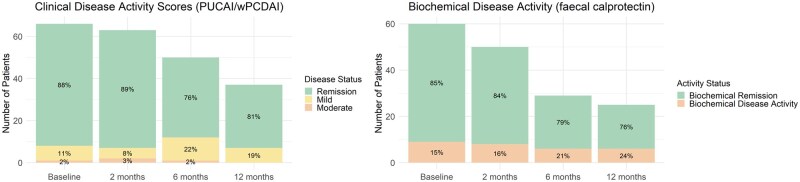
Trends in clinical disease activity scores and biochemical disease activity.

At time of switch, 58/66 patients (88%) were in clinical remission and FC level was <250 µg/g in 51/60 patients (85%), with a median FC level of 45.5 µg/g (IQR 25-119 µg/g). During follow-up, the proportion of patients in clinical remission was 56/63 (89%) at 2 months after start of SC-IFX, 38/50 (76%) at 6 months and 30/37 (81%) at 12 months. Similarly, the proportion of patients in biochemical remission was 42/50 (84%), 23/29 (79%) and 19/25 (76%) respectively. Clinical disease activity and FC levels during follow-up are presented in Figure 2.

### Infliximab levels and immunogenicity

**Figure 3:**
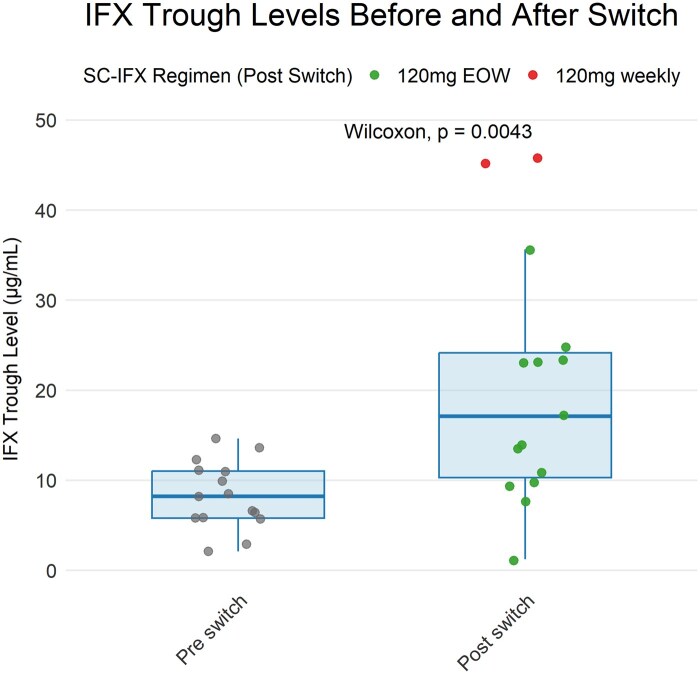
Sub-analysis of IFX (trough) levels before and after switch to SC-IFX of patients where the exact (trough) level was determined during IV-IFX treatment and 2 months after switch. Abbreviations: IFX, infliximab; IV-IFX, intravenous infliximab; SC-IFX, subcutaneous infliximab.

Median IFX (trough) levels pre-switch and at 2, 6 and 12 months after switch to SC-IFX were 12.0 (IQR 9.5-12.0; n = 59), 18.8 (IQR 14.0-24.2; n = 56), 14.0 (12.0-17.6; n = 22) and 12.0 µg/mL (IQR 11.9-14.4; n = 20) respectively. A sub-analysis of patients for whom IFX (trough) levels were available without approximation due to assay ceiling effects, demonstrated a significant increase after switching to SC-IFX from 8.2 µg/mL (IQR 5.8-11.0) to 17.1 µg/mL (IQR 10.3-24.2) (Figure 3**, *P* = 0.004**). This increase remained significant after excluding 2 patients switched to a regimen of SC-IFX 120 mg EW (6.6 µg/mL (IQR 5.8-11.1) to 13.9 µg/mL (IQR 9.8-23.0) ***P* = 0.02**). At time of switch, 4/66 patients (6%) had detectable ADA of 9, 16, 128 and 149 ng/mL: one of these patients’ ADA titre increased after switch to SC-IFX from 149 to 292 ng/mL and subsequently discontinued ­treatment, while the remaining 3 showed resolution of ADA levels after switching, without initiation of additional co-immunosuppression. During follow-up, 2/66 patients (3%) developed ADA: one patient had a titre of 83 ng/mL, however transferred to adult care was lost to follow-up, and one had a titre of 11 ng/mL and showed spontaneous normalization. In total, one patient had immunogenic LOR with an IFX level of 1.2 µg/mL and ADA titre of 292 ng/mL and discontinued SC-IFX treatment.

At baseline, 26 patients (39%) were using a concomitant immunomodulator (Table 1). During follow-up, 9/26 patients (35%) discontinued co-immunosuppression. Of these, 7 were able to stop co-immunosuppression uneventfully; one patient developed a relapse and one patient, who was not in remission at time of switch, did not reach remission during follow-up. None of the patients who discontinued co-immunosuppression developed ADA.

### Specific sub cohorts

Of the 17 patients previously on a 10 mg/kg 4 weekly IV-IFX regimen, one patient switched directly to the 120 mg EW SC-IFX regimen. During follow-up, 2 additional patients required dose intensification, resulting in 3 out of 17 patients (18%) receiving 120 mg EW. The remaining patients were maintained on SC-IFX 120 mg EOW. There were no differences in treatment persistence, relapse rate, AEs, SC-IFX dosing regimen and IFX levels post-switch (Table S2).

Eleven out of 66 patients (17%) weighed 80 kg or more at time of switch, with a median BMI of 32.5 (IQR: 29.1-33.3). Interestingly, IFX levels 2 months post-switch to SC-IFX were lower in patients weighing > 80kg compared to ≤ 80kg, with median levels of 14 and 20 µg/mL (*P* = .008). In addition, AEs were higher in the group ≤ 80kg with 19/55 and 0/11 AEs respectively (*P* = .026) (Table S3). Six out of 66 patients (9%) weighed 40 kg or less at time of switch. There were no differences between patients weighing ≤ 40kg and > 40kg in treatment persistence, relapse rate, AEs, dosing regimen, and IFX levels (Table S4).

Two patients switched to SC-IFX directly following 3 infusions of IV-IFX induction therapy. Neither patient was in remission at time of switch, however, both achieved clinical remission by the 2-month follow-up. Both patients were started on SC-IFX 120 mg EOW. Notably, neither patient experienced a relapse or discontinued SC-IFX treatment.

### Safety

Adverse events (AEs) were reported in 19/66 patients (29%), with 21 AEs reported in total during follow-up. The most frequently reported AEs were pain or swelling at injection site (7/66, 11%) and new-onset or worsening of eczema (6/66, 9%). Three patients (3/66, 5%) experienced serious AEs requiring hospitalization, namely IgA-mediated nephropathy, pyelonephritis requiring IV antibiotics, and perianal fistula requiring seton drainage. No deaths or malignancies occurred. A full characterization of AEs is provided in Table 4.

### Patient acceptance and satisfaction

Of the 66 patients included, 53 (80%) expressed a positive attitude toward the use of SC-IFX. Eleven out of 66 patients (17%) reported challenges related to SC-IFX administration. The most common issue was injection-site pain (*n* = 6), followed by anticipatory anxiety (*n* = 2), difficulty with self-administration (*n* = 1), and urticaria post-injection (*n* = 1). One patient expressed a general dislike for SC-IFX without specifying the reason.

### Economic and environmental impact

Maintenance treatment with SC-IFX does not result in an overall increase in total estimated costs compared to IV-IFX, although there is high inter-country variability in these costs. Switching to SC-IFX would result in an estimated greenhouse gas emission reduction of 45.88 kg CO2e per year per patient (Supplemental Content 1).

## Discussion

In what is to our knowledge the largest currently published and first international multicenter real-world cohort of PIBD patients who switched from IV-IFX to SC-IFX, we observed high treatment persistence up to 1-year post-switch, with a minority of patients requiring SC-IFX dose escalation and few a switch back to IV-IFX. Clinical and biochemical disease stability was maintained post-switch in this cohort, the majority of whom were in clinical and biochemical remission on a stable IV-IFX regimen at the time of switch.

To date, only 2 case series have been published on SC-IFX in PIBD. The first included 7 patients, all of whom are incorporated within the present cohort.^20^ The second included 21 patients, all in clinical remission at the time of switch, who maintained remission over six months of follow-up without any discontinuations.^21^ Given the paucity of pediatric evidence, adult data provide the main realistic comparator; however, important differences must be acknowledged, including the markedly higher proportion of pediatric patients on intensified IV-IFX regimens before switching, the wide variability in body weight intrinsic to pediatric populations, and the weight/age-related differences in pharmacokinetics.

Studies of real-world adult cohorts, which have predominantly assessed SC-IFX switch during stable IV-IFX maintenance and only rarely included direct comparisons of IV and SC arms, have consistently shown that SC-IFX is largely comparable to IV-IFX in terms of efficacy and safety,^8–11^^,^^13^^,^^17^^,^^25–28^ with a recent meta-analysis suggesting a potential clinical benefit of the subcutaneous formulation.^18^

In our study, the 12-month SC-IFX discontinuation-free survival rate was 78% (95% CI, 66%-91%) and the overall persistence on infliximab—either SC or IV—was in 89%. These findings align with adult real-world data, in which SC-IFX persistence at 6-12 months ranges from 67% to 95% in patients mostly switched to SC-IFX while in stable remission.^8–11^^,^^13^^,^^25–28^ Reported LOR-rated SC-IFX discontinuations in adults (3%-4% at 12-18 months post-switch) are comparable to our observations (3/66, 5%).^9^^,^^10^

Disease relapse was experienced by 17% of our patients, with clinical remission rates ranging between 76% and 89% over a median follow-up of 11 months post SC-IFX-switch. These findings are consistent with adult SC-IFX data, where clinical remission rates at 6-12 months post-switch range between 69% and 97%^8–11^^,^^13^^,^^16^^,^^25–28^ and a pooled median relapse rate of 11% (IQR 6%-16%) at 12 months has been reported in patients mostly switched while in stable remission.^29^ Comparable long-term remission rates are also observed with IV-IFX, as shown in the PANTS study, where the estimated proportion of patients in remission at 2 and 3 years after IV-IFX initiation was 71% (95% CI, 63%-78%) and 63% (95% CI, 55%-71%), respectively, among those already in remission at year 1.^30^

Several baseline factors were examined in our exploratory analysis as predictors of both SC-IFX discontinuation and relapse. Multivariate analysis identified higher clinical scores at switch as the only independent factor significantly associated with relapse risk over time (HR, 1.05; 95% CI, 1.00-1.11; *P* = .04). This result aligns with part of adult evidence suggesting that switching during active disease is associated with higher relapse rates compared to switching during remission. In the retrospective study by D’Amico et al., patients who switched to SC-IFX at week 6 were compared with those who switched later on. Early switchers showed higher rates of therapeutic changes at 3, 6, and 12 months, and baseline clinical disease activity in UC was identified as a risk factor for discontinuation, optimization, steroid need, and reverse switch.^13^ Conversely, in the study by Bertani et al. early switch to SC-IFX at week 6 was as effective as late switch at 6 months in terms of 1-year clinical and endoscopic remission, with neither clinical nor biochemical disease activity scores predictive of outcome.^27^

In contrast with part of adult literature, an intensified IV-IFX regimen (10 mg/kg every 4 weeks) pre-switch was not found to be significantly associated with unfavorable outcomes. In the REMSWITCH study, Buisson et al. reported that switching from IV-IFX 10 mg/kg every 4 weeks) was significantly associated with an increased risk of relapse; thus, the authors proposed that these patients should be considered for higher SC-IFX regimens, such as 240 mg EOW.^9^ This approach was also suggested by the French pediatric case series, where 3 of 8 patients on IV-IFX 10 mg/kg every 4 weeks escalated to 240 mg EOW after SC-IFX switch, although this did not translate into differences in clinical remission or discontinuation rates.^21^ However, our findings, along with other adult real-world cohorts such as the French study by Mathieu et al. did not confirm these associations, supporting the feasibility of a 120 mg EOW starting regimen regardless of the preceding IV-IFX dosing schedule, with the possibility of successful dose intensification in cases of relapse.^11^

Baseline weight, which is subject to considerable variability across the pediatric age range, was not predictive of relapse or treatment discontinuation. Unlike the French pediatric series, where all patients >80 kg were switched to SC-IFX 240 mg EOW, all >80-kg patients in our cohort (*n* = 11) were switched to 120 mg EOW, with only 2/11 requiring intensification to weekly dosing.^21^ Our findings in patients >80 kg mirror those of adult overweight/obese cohorts, in whom BMI has not consistently been associated with poorer outcomes after switching to SC-IFX, despite being linked to lower infliximab levels post-switch.^11^^,^^26^^,^^32^^,^^33^ A distinctive feature of our cohort is the inclusion of 6 patients weighing <40 kg, with relapse, discontinuation, and AE rates comparable to those in patients weighing  ≥40 kg. Although a simulation study predicted higher SC-IFX exposure in children compared to adults,^34^ this theoretical model lacked pediatric SC-IFX pharmacokinetic data. Absorption characteristics may therefore differ, and pediatric-specific pharmacokinetic studies are needed to define optimal SC-IFX dosing. This is particularly relevant given the known higher IFX clearance in younger children and the proven limitations of extrapolating adult dosing to lower body weights.^35^

Our pharmacokinetic analysis, although limited by assay constraints, demonstrated a significant rise in IFX levels after switching to SC-IFX. The CT-P13 SC 1.6 trial as well as all real-world adult studies have similarly reported higher median SC-IFX levels post-switch.^6^^,^^11^^,^^26^^,^^26^ However, levels do not directly reflect total drug exposure, with area under the curve (AUC) being a more appropriate parameter.^6^^,^^36^ Nevertheless, therapeutic drug monitoring may serve as a surrogate marker, although a careful interpretation of these elevated SC-IFX levels is required, avoiding direct analysis according to IV-IFX therapeutic ranges.^31^^,^^36^ Few studies have proposed SC-specific thresholds. In the ENEIDA study, a week-52 SC-IFX level >13 µg/mL predicted clinical and biochemical remission, whereas Roblin et al. identified >20 µg/mL for deep remission.^16^^,^^37^

As a direct consequence of the different administration route, compared to IV-IFX, SC-IFX provides higher and more stable serum concentrations, avoiding peak-trough variability, potentially lowering immunogenicity and offering greater flexibility in TDM.^6^^,^^36^^,^^38^ In the CT-P13 1.6 trial, similar proportions of ADA-positive patients were observed in the SC-IFX and IV-IFX arms throughout the study; however, a significantly lower proportion of patients developed neutralizing antibodies in the SC-IFX group.^6^ Subsequent real-world studies confirmed low immunogenicity rates on SC-IFX and even successful SC-IFX use after prior immunogenic failure to IV-IFX.^11^^,^^26^^,^^39^ Although in our cohort inconsistent ADA detection limits firm conclusions, 3 of 4 ADA-positive patients at switch reverted to negative after SC-IFX start.

These data have prompted reconsideration of the role of concomitant immunosuppression with SC-IFX. In our study, no patients who discontinued co-immunosuppression developed ADA, and 67% (6/9) did so uneventfully. A recent meta-analysis reported higher ADA rates with SC monotherapy than with combination therapy 1 year post-switch.^29^ However, interpretation is limited by methodological heterogeneity among the studies considered in the metanalysis, including the use of drug-tolerant vs drug-sensitive ADA assays and variability in the timing of the switch. Such limitations warrant cautious interpretation, particularly since differences in ADA rates between SC-IFX monotherapy and combination therapy have not been consistently associated with clinical failure.^29^ Given potential safety advantages, further studies should clarify whether combination therapy remains necessary with SC-IFX.

From a safety perspective, our findings support the favorable profile of SC-IFX observed in adult studies. AEs were reported in 19/66 patients (29%), totaling 21 events, most commonly injection-site reactions or new/worsening eczema (13/21, 62%), while serious AEs were infrequent (3/66, 5%). These frequencies are consistent with adult data from pivotal CT-P13 SC trials, where treatment-emergent and serious AEs were comparable between the IV and SC arms (AEs, 58% vs 49%; serious AEs, 3% vs 4%), though injection-site reactions were, as expected, more common with SC treatment.^6^

Switching practices in our cohort evolved over time. Early switches were largely patient driven, prompted by factors such as needle phobia, difficult intravenous access, or lifestyle preferences. With growing clinical experience, switching became part of a structured program, aimed primarily, though not exclusively, at patients in stable remission and implemented through shared decision-making between clinicians and families. Consistent with this patient-centered approach, most patients in our study (80%) expressed a positive attitude toward SC-IFX, in line with adult data showing high satisfaction and preference for the SC formulation.^8^^,^^40^ Nevertheless, careful monitoring by the IBD team remains essential to ensure treatment adherence during and after the switch. In our cohort, most discontinuations (64%) were related to difficulties with the SC route, highlighting the importance of identifying the most suitable candidates. In this regard, it is relevant that switching back to IV-IFX remains a safe option. In the largest available adult cohort, Bothorel et al. reported that switching back to IV-IFX was highly effective in patients dissatisfied with SC-IFX, with 96% of those discontinuing SC therapy successfully reverting to IV-IFX.^15^ These findings support the flexibility of switching strategies, while emphasizing the need for individualized follow-up and shared decision-making. The main limitations of our study include its retrospective design and relatively small sample size which, although smaller than typical adult cohorts, remains substantial for a pediatric population. The low number of events preclude more robust analyses of predictors of relapse or discontinuation and the absence of a comparative IV-IFX population prevents a direct comparison between the 2 routes of IFX administration. Furthermore, our evaluation primarily focused on biochemical disease outcomes, as post-switch monitoring was mainly directed toward these parameters, resulting in less systematic assessment of perianal disease activity. The pharmacokinetic sub-analysis was also constrained by methodological limitations, particularly a ceiling effect in the assay used. Prospective pediatric studies with specific pharmacokinetic analyses are needed to extend treatment into the <40-kg population.

Despite these limitations, our study has important strengths. It represents the first multicenter pediatric cohort investigation providing meaningful insights beyond isolated case series and allowing meaningful comparison with adult experience. Moreover, the real-world nature of this study, encompassing a broad range of clinical scenarios, reflects the variability of everyday pediatric practice.

In conclusion, in what is to our knowledge the largest pediatric real-world cohort described to date, SC-IFX proved safe, effective, and well accepted as a maintenance therapy, with low immunogenicity and high treatment persistence. Standard dosing appeared feasible regardless of baseline characteristics, including intensified infliximab regimens, with the option for SC-IFX dose escalation in the event of disease relapse. These findings support wider implementation of SC-IFX, offering reduced treatment burden for patients and families and decreased infusion demand for health care systems. Future studies are needed to define optimal dosing across weight and age ranges in pediatric patients, particularly in children weighing <40 kg, and to work toward specific licensing within the pediatric population.

## Supplementary Material

izaf335_Supplementary_Data
