# Supplementary material for: Persistence and safety of subcutaneous infliximab up to 1 year after switching from intravenous infliximab in pediatric inflammatory bowel disease: a multicenter real-world cohort study
Source: Inflamm Bowel Dis. 2026 Feb 1;32(6):1086–96. doi: 10.1093/ibd/izaf335 (PMC13233139; doi:10.1093/ibd/izaf335)
Supplement: izaf335_Supplementary_Data [file izaf335_supplementary_data.docx]

# Supplementary Material

| **Characteristic** | **Edinburgh**  N = 37*^1^* | **Rotterdam**  N = 29*^1^* | **p-value***^2^* |
| --- | --- | --- | --- |
| Diagnosis, n(%) |  |  | 0.3 |
| Crohn's Disease | 21 (57%) | 20 (69%) |  |
| IBD Unclassified | 3 (8%) | 0 (0%) |  |
| Ulcerative Colitis | 13 (35%) | 9 (31%) |  |
| Sex, n(%) |  |  | 0.8 |
| Female | 20 (54%) | 14 (48%) |  |
| Male | 17 (46%) | 5 (52%) |  |
| Weight at switch, kg, median (min-max) | 61 (38.2, 102) | 57 (13.0, 98.0) | 0.054 |
| Weight, z-score, mean (SD) | 0.26 (1.23) | 0.64 (1.3) | 0.2 |
| Height, z-score, mean (SD) | -0.08 (1.11) | -0.21 (1.0) | 0.6 |
| BMI at switch, mg/kg^2^, median(IQR) | 21.7 (19.5, 24.2) | 20.6 (17.8, 23.2) | 0.2 |
| Disease duration at switch, months, median(IQR) | 38 (20, 53) | 27 (13, 36) | 0.12 |
| Age at switch, years, median(IQR) | 16.83 (16.3, 17.6) | 15.1 (12.9, 16.7) | 0.001 |
| Paris age classification at diagnosis, n(%) |  |  | 0.045 |
| A1a | 1 (5%) | 6 (30%) |  |
| A1b | 15 (100%) | 15 (71%) |  |
| Paris disease behaviour at diagnosis, n(%) |  |  | 0.8 |
| B1 | 18 (86%) | 16 (80%) |  |
| B2 | 2 (9.5%) | 2 (10%) |  |
| B3 | 1 (4.5%) | 2 (10%) |  |
| Paris disease location at diagnosis, n(%) |  |  | 0.5 |
| L1 | 1 (5%) | 4 (20%) |  |
| L2 | 4 (19%) | 3 (15%) |  |
| L3 | 15 (71%) | 13 (65%) |  |
| L4 | 1 (5%) | 0 (0%) |  |
| Growth restriction at diagnosis, n(%) |  |  | >0.9 |
| G0 | 18 (86%) | 17 (85%) |  |
| G1 | 3 (14%) | 3 (15%) |  |
| Perianal disease at diagnosis, yes, n(%) | 6 (29%) | 5 (25%) | >0.9 |
| Paris disease extent at diagnosis, n(%) |  |  | 0.5 |
| E1 | 0 (0%) | 1 (11%) |  |
| E2 | 2 (13%) | 2 (22%) |  |
| E3 | 5 (31%) | 1 (11%) |  |
| E4 | 9 (56%) | 5 (56%) |  |
| Disease severity at diagnosis, n(%) |  |  | 0.2 |
| S0 | 6 (38%) | 6 (67%) |  |
| S1 | 10 (63%) | 3 (33%) |  |
| Previous surgery, yes, n(%) | 1 (2.7%) | 4 (14%) | 0.2 |
| Previous mesalazine, yes, n(%) | 12 (32%) | 9 (31%) | >0.9 |
| Previous immunomodulator, yes, n(%) | 32 (86%) | 29 (100%) | 0.062 |
| Previous corticosteroids, yes, n(%) | 26 (70%) | 16 (55%) | 0.3 |
| Previous adalimumab, yes, n(%) | 4 (11%) | 1 (3%) | 0.4 |
| Previous intravenous infliximab, yes, n(%) | 37 (100%) | 29 (100%) |  |
| Duration intravenous infliximab therapy, months, n(%) | 26 (13, 37) | 21 (7, 32) | 0.2 |
| Intravenous infliximab interval, weeks, n(%) |  |  | 0.3 |
| 4 | 13 (35%) | 5 (17%) |  |
| 5 | 1 (3%) | 1 (3%) |  |
| 6 | 16 (43%) | 14 (48%) |  |
| 8 | 7 (19%) | 7 (24%) |  |
| Only induction scheme | 0 (0%) | 2 (7%) |  |
| Intravenous infliximab dose, mg/kg | 10.00 (10.0, 10.0) | 10.00 (6.3, 10.0) | 0.003 |
| Intravenous infliximab regimen 10mg/kg 4 weekly, yes, n(%) | 13 (35%) | 4 (14%) | 0.049 |
| Concomitant immunosuppression at switch, yes, n(%) | 22 (59%) | 4 (14%) | <0.001 |
| Subcutaneous infliximab regimen, n(%) |  |  | 0.033 |
| 120mg, 2 weekly | 37 (100%) | 25 (86%) |  |
| 120mg, weekly | 0 (0%) | 4 (14%) |  |
| Persistence on subcutaneous infliximab, yes, n(%) | 33 (89%) | 22 (76%) | 0.2 |
| Adverse events, yes, n(%) | 9 (24%) | 10 (34%) | 0.4 |
| Clinical remission at inclusion, yes, n(%) | 35 (95%) | 23 (79%) | 0.12 |
| PUCAI at switch, median(IQR) | 0.0 (0.0, 0.0) | 0.0 (0.0, 5.0) | 0.049 |
| Missing | 0 | 1 |  |
| wPCDAI at switch, median(IQR) | 0.0 (0.0, 0.0) | 0.0 (0.0, 7.5) | 0.5 |
| Faecal calprotectin at switch, median(IQR) | 45 (25, 85) | 56 (20, 324) | 0.7 |
| Missing | 2 | 4 |  |
| CRP at switch, median(IQR) | 1.0 (1.0, 1.0) | 0.8 (0.6, 3.0) | 0.6 |
| ESR at switch, median(IQR) | 8 (4, 10) | 13 (8, 23) | 0.013 |
| Missing | 0 | 5 |  |
| Albumin at switch, median(IQR) | 39.0 (37.0, 41.0) | 40.0 (37.0, 42.0) | 0.2 |
| Infliximab trough level during intravenous infliximab, µg/ml, median(IQR) | 12.0 (11.5, 12.0) | 10.5 (7.4, 19.2) | 0.8 |
| Missing | 0 | 7 |  |
| *^1^*n (%); Mean (SD); Median (Q1, Q3) | | | |
| *^2^*Fisher's exact test; Welch Two Sample t-test; Mann Whitney U test; Pearson's Chi-squared test | | | |

**Supplementary Table 1:** Baseline characteristics of patients at time of initiation with subcutaneous infliximab stratified by hospital.


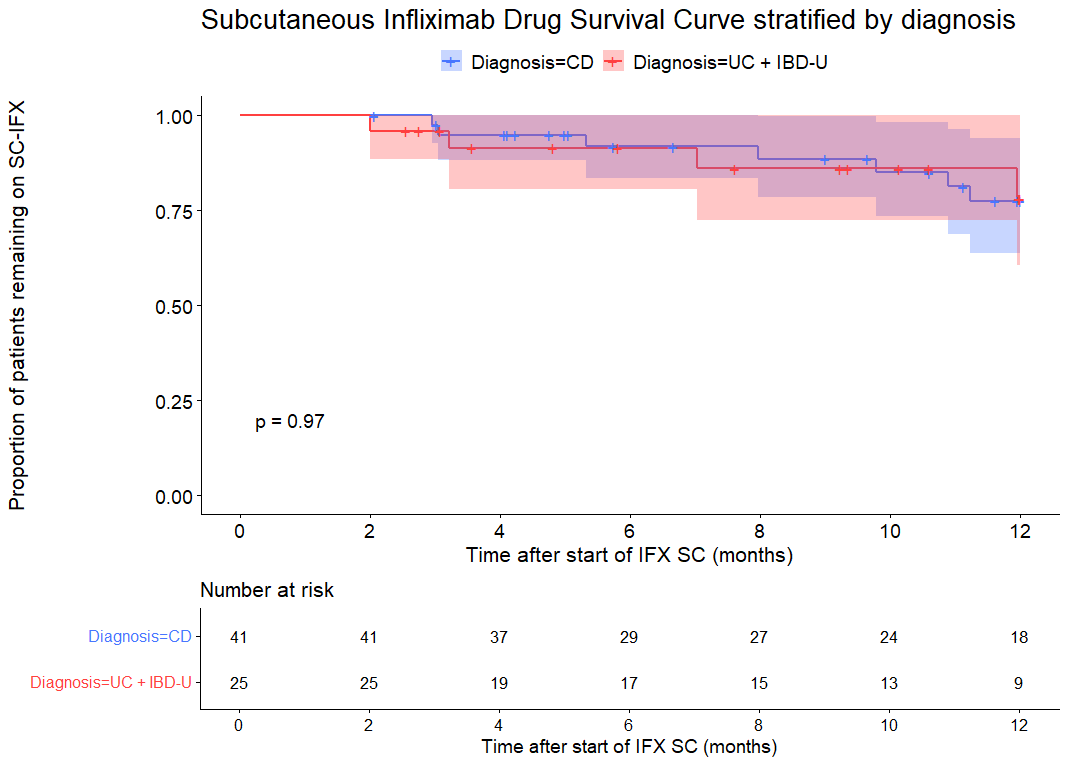


**Supplementary Figure 1:** Kaplan-Meier curve of treatment persistence on subcutaneous infliximab stratified by diagnosis.

| **Characteristic** | **No intensified IV-IFX regimen**  N = 49*^1^* | **Intensified IV-IFX regimen** N = 17*^1^* | **p-value***^2^* |
| --- | --- | --- | --- |
| Diagnosis, n(%) |  |  | >0.9 |
| Crohn's Disease | 31 (63%) | 10 (59%) |  |
| Disease duration at switch, months, median(IQR) | 34 (21, 50) | 24 (16, 52) | 0.5 |
| Age at switch, years, median(IQR) | 16.59 (15.11, 17.33) | 16.17 (14.67, 16.93) | 0.4 |
| Duration intravenous infliximab therapy, months, n(%) | 26 (13, 39) | 17 (8, 24) | 0.072 |
| Subcutaneous infliximab regimen, n(%) |  |  | >0.9 |
| 120mg, 2 weekly | 46 (94%) | 16 (94%) |  |
| Clinical remission at inclusion, yes, n(%) | 43 (88%) | 15 (88%) | >0.9 |
| Faecal calprotectin at switch, median(IQR) | 38 (23, 100) | 54 (25, 178) | 0.4 |
| Missing | 5 | 1 |  |
| Infliximab trough level during intravenous infliximab, µg/ml, median(IQR) | 11.8 (9.0, 12.0) | 12.0 (12.0, 12.0) | 0.050 |
| Missing | 7 | 0 |  |
| Infliximab level 2 months post-switch, µg/ml, median(IQR) | 20 (14, 24) | 16 (12, 25) | 0.3 |
| Missing | 7 | 3 |  |
| Persistence on subcutaneous infliximab, yes, n(%) | 42 (86%) | 13 (76%) | 0.5 |
| Adverse reaction, yes, n(%) | 16 (33%) | 3 (18%) | 0.4 |
| Relapse, yes, n(%) | 8 (16%) | 3 (18%) | >0.9 |
| *^1^*n (%); Mean (SD); Median (Q1, Q3) | | | |
| *^2^*Fisher's exact test; Welch Two Sample t-test; Mann Whitney U test; Pearson's Chi-squared test | | | |

**Supplementary Table 2**: Characteristics of patients on subcutaneous infliximab stratified by intensified IV-IFX regimen yes/no.

| **Characteristic** | **≤80kg**  N = 55*^1^* | **>80kg** N = 11*^1^* | **p-value***^2^* |
| --- | --- | --- | --- |
| Diagnosis, n(%) |  |  | 0.5 |
| Crohn's Disease | 35 (64%) | 6 (55%) |  |
| Disease duration at switch, months, median(IQR) | 34 (19, 53) | 27 (17, 40) | 0.6 |
| Age at switch, years, median(IQR) | 16.50 (14.27, 17.33) | 16.92 (16.17, 17.58) | 0.2 |
| Duration intravenous infliximab therapy, months, n(%) | 24 (10, 36) | 19 (10, 34) | 0.5 |
| Intravenous infliximab regimen 10mg/kg 4 weekly, yes, n(%) | 12 (22%) | 5 (45%) | 0.13 |
| Subcutaneous infliximab regimen, n(%) |  |  | >0.9 |
| 120mg, 2 weekly | 51 (93%) | 11 (100%) |  |
| Clinical remission at inclusion, yes, n(%) | 47 (85%) | 11 (100%) | 0.3 |
| Faecal calprotectin at switch, median(IQR) | 46 (20, 115) | 51 (25, 131) | 0.6 |
| Missing | 5 | 1 |  |
| Infliximab trough level during intravenous infliximab, µg/ml, median(IQR) | 12.0 (9.8, 12.2) | 12.0 (7.4, 12.0) | 0.6 |
| Missing | 7 | 0 |  |
| Infliximab level 2 months post-switch, µg/ml, median(IQR) | 20 (14, 25) | 14 (10, 15) | 0.008 |
| Missing | 9 | 1 |  |
| Persistence on subcutaneous infliximab, yes, n(%) | 47 (85%) | 8 (73%) | 0.4 |
| Adverse reaction, yes, n(%) | 19 (35%) | 0 (0%) | 0.026 |
| Relapse, yes, n(%) | 10 (18%) | 1 (9.1%) | 0.7 |
| *^1^*n (%); Mean (SD); Median (Q1, Q3) | | | |
| *^2^*Fisher's exact test; Welch Two Sample t-test; Mann Whitney U test; Pearson's Chi-squared test | | | |

**Supplementary Table 3**: Characteristics of patients on subcutaneous infliximab stratified by weight ≤80kg and >80kg.

| **Characteristic** | **≤40kg**  N = 6*^1^* | **>40kg** N = 60*^1^* | **p-value***^2^* |
| --- | --- | --- | --- |
| Diagnosis, n(%) |  |  | 0.7 |
| Crohn's Disease | 5 (83%) | 36 (60%) |  |
| Disease duration at switch, months, median(IQR) | 20 (10, 23) | 35 (20, 53) | 0.031 |
| Age at switch, years, median(IQR) | 10.40 (6.93, 13.75) | 16.59 (15.34, 17.46) | <0.001 |
| Duration intravenous infliximab therapy, months, n(%) | 9 (7, 21) | 25 (11, 37) | 0.029 |
| Intravenous infliximab regimen 10mg/kg 4 weekly, yes, n(%) | 3 (50%) | 14 (23%) | 0.2 |
| Subcutaneous infliximab regimen, n(%) |  |  | 0.3 |
| 120mg, 2 weekly | 5 (83%) | 57 (95%) |  |
| Clinical remission at inclusion, yes, n(%) | 5 (83%) | 53 (88%) | 0.6 |
| Faecal calprotectin at switch, median(IQR) | 23 (20, 25) | 49 (25, 131) | 0.12 |
| Missing | 0 | 6 |  |
| Infliximab trough level during intravenous infliximab, µg/ml, median(IQR) | 11.6 (9.6, 19.2) | 12.0 (9.4, 12.0) | >0.9 |
| Missing | 0 | 7 |  |
| Infliximab level 2 months post-switch, µg/ml, median(IQR) | 25 (17, 33) | 17 (14, 24) | 0.3 |
| Missing | 2 | 8 |  |
| Persistence on subcutaneous infliximab, yes, n(%) | 4 (67%) | 51 (85%) | 0.3 |
| Adverse reaction, yes, n(%) | 2 (33%) | 17 (28%) | >0.9 |
| Relapse, yes, n(%) | 1 (17%) | 10 (17%) | >0.9 |
| *^1^*n (%); Mean (SD); Median (Q1, Q3) | | | |
| *^2^*Fisher's exact test; Welch Two Sample t-test; Mann Whitney U test; Pearson's Chi-squared test | | | |

**Supplementary Table 4**: Characteristics of patients on subcutaneous infliximab stratified by weight ≤40kg and >40kg.

**Supplemental content 1**

**Methods**

*Economic and environmental impact*

To estimate the economic impact of the switching programme to SC-IFX, both direct and indirect costs were considered for both cohorts. For the Netherlands, a general market discount of 60% applied to biosimilars was used to calculate the price. As direct costs we considered for IV-IFX the cost of a single 100mg biosimilar vial was used, priced at €78 in the United Kingdom and €145.20 in the Netherlands. For SC-IFX, the cost of a pack of two pre-filled pens was taken for the United Kingdom, priced at €367, and for the Netherlands, the cost of a single pre-filled pen was €140.38. These costs were multiplied by the median dose, frequency of use, and the weight (for IV-IFX) of the population. Moreover, for IV-IFX the non-drug administration costs (staff, equipment and infusion unit standard tariffs) were €153,40 per patient per visit as per locally agreed costs for the Edinburgh cohort. Costs related to outpatient clinic visits were left out of the calculation, as these were expected to be similar between IV-IFX and SC-IFX. For the Rotterdam cohort, non-drug administration costs were not explicitly determined. However, the difference in total costs can be inferred from the difference in the Diagnosis Treatment Combination (DBC; Dutch bundled care tariff). The DBC for 1–2 IV-IFX infusions every 120 days is €1,826.29, and for more than 2 infusions it is €3,522.25. For SC-IFX, based on outpatient clinic visits, the DBC is €421.48 for 1–2 visits and €1,826.29 for more than 2 visits per 120 days.

For the indirect costs, both occupational and travel costs were considered. Occupational costs were estimated based on the fact that all patients were accompanied by a working parent. Using national statistics, the cost of a working day lost was calculated as €143.10 in Scotland (from the Annual Survey for Hours and Earnings (ASHE)) (1), and €153.88 in the Netherlands (from the Dutch National Statistical Office) (2). For travel costs, since the car was the most commonly used mode of transportation, an estimate was made by selecting the most frequently used car in both Scotland and the Netherlands in 2024, which was petrol-powered (3, 4). The petrol rate was assumed to be €0.102 per kilometre for cars with an engine size between 1401-2000cc (5). Travel and time distances were evaluated via Google Maps for round-trips from patient’s address to the hospital on a weekday.

As secondary outcome the environmental impact of the switch in terms of kilometres saved and reduction of greenhouse gas (GHGs) emissions was considered. GHGs were calculated via the conversion factors used by UK organization to report on 2020 GHG emissions; car emissions were estimated using the category ‘‘average car’’ and ‘‘petrol fuel’’ (1 kilometre = 0.174 kgCO2e) (6).

**Results**

*Economic impact*

Considering the median IV-IFX maintenance dose (10mg/kg every 6 weeks) and the average weight of 60kg in our population, the drug-related direct costs for IV-IFX were estimated at €468.00 per infusion in Scotland and €871.20 per infusion in The Netherlands. For SC-IFX, on an equal 6-weekly basis, the drug-related direct costs were estimated at €550.50 in Scotland and €421.14 in The Netherlands. In addition, the direct non-drug-related costs per patient per year in Scotland were estimated at €1329.67 for IV-IFX. When combining these, the total estimated costs per patient per year for IV-IFX were €5385.67and for SC-IFX were €4771.00 in Scotland. For The Netherlands, the total direct non-drug related costs per patient per year was €8870.79 euros for IV-IFX and €1264.44 for SC-IFX (based on 1-2 outpatient visits every 120 days), which combined leads to an estimate of total direct costs of €16421.19 for IV-IFX and €4914.21 for SC-IFX per year. Occupational costs, in terms of working days lost, were estimated at €1240.20 for IV-IFX and €572.40 for SC-IFX in Scotland, and €1333.63 for IV-IFX and €615.52 for SC-IFX in The Netherlands. Travel costs were calculated based on a median distance of 28.25 km from the hospital, with IV-IFX resulting in €49.95 per patient per year, and SC-IFX at €23.05 per year. Additionally, the median travel time for a single hospital visit was estimated at 67 minutes per patient, leading to 580.67 minutes of total travel time per year for IV-IFX and 268 minutes for SC-IFX.

The total direct and indirect estimated costs for IV-IFX for a patient in Scotland counts up to €5385.67 per patient per year and for a patient in the Netherlands €16421.19. The total estimated costs for SC-IFX are €4771.00 for Scotland and €4914.21 per year for The Netherlands.

*Environmental impact*

From an environmental perspective, considering a median distance from the hospital 28.25 kilometres, SC-IFX requires travel of 226 kilometres per year and IV-IFX requires travel of 489.67 kilometres per year. Consequently, the amount of GHG emissions are estimated to be 39.32 kg CO2e per patient per year on SC-IFX and 85.20 kg CO2e per patient per year.

**References**:

1. Office of National Statistics. Statistical bulletin. Employee earnings in the UK: 2024.

https://www.ons.gov.uk/employmentandlabourmarket/peopleinwork/earningsandworkinghours/datasets/allemployeesashetable1. Published October 29, 2024. Accessed August 7, 2025

2. Centraal Bureau van Statistiek. Inkomen van personen; inkomensklassen, persoonskenmerken. <https://opendata.cbs.nl/statline/#/CBS/nl/dataset/83931NED/table?dl=D4D1>. Published November 1, 2024. Accessed August 7, 2025.

3. Transport Scotland. Scottish Transport Statistics 2024. https://www.transport.gov.scot/publication/scottish-transport-statistics-2024/. Published March 25, 2025. Accessed August 8, 2025

4. Kerncijfers Auto en Mobiliteit. <https://www.bovag.nl/pers/cijfers>. Published 2025. Accessed August 8, 2025.

5. GOV.UK. Advisory fuel rates 2025. https://www.gov.uk/guidance/advisory-fuel-rates. Published May 22, 2025. Accessed August 8, 2025

6. GOV.UK. Greenhouse gas reporting: conversion factors https://www.gov.uk/government/publications/greenhouse-gas-reporting-conversion-factors-2020. Published June 9, 2020. Accessed August 8, 2025
